# Tropical parabiotic ants: Highly unusual cuticular substances and low interspecific discrimination

**DOI:** 10.1186/1742-9994-5-16

**Published:** 2008-10-20

**Authors:** Florian Menzel, Nico Blüthgen, Thomas Schmitt

**Affiliations:** 1 University of Würzburg, Biocenter, Department of Animal Ecology and Tropical Biology, Am Hubland, 97074 Würzburg, Germany; 2 University of Freiburg, Institute of Biology I (Zoology), Department of Evolutionary Biology and Animal Ecology, Hauptstr.1, 79104 Freiburg, Germany

## Abstract

**Background:**

Associations between animal species require that at least one of the species recognizes its partner. Parabioses are associations of two ant species which co-inhabit the same nest. Ants usually possess an elaborate nestmate recognition system, which is based on cuticular hydrocarbons and allows them to distinguish nestmates from non-nestmates through quantitative or qualitative differences in the hydrocarbon composition. Hence, living in a parabiotic association probably necessitates changes of the nestmate recognition system in both species, since heterospecific ants have to be accepted as nestmates.

**Results:**

In the present study we report highly unusual cuticular profiles in the parabiotic species *Crematogaster modiglianii *and *Camponotus rufifemur *from the tropical rainforest of Borneo. The cuticle of both species is covered by a set of steroids, which are highly unusual surface compounds. They also occur in the Dufour gland of *Crematogaster modiglianii *in high quantities. The composition of these steroids differed between colonies but was highly similar among the two species of a parabiotic nest. In contrast, hydrocarbon composition of *Cr. modiglianii *and *Ca. rufifemur *differed strongly and only overlapped in three regularly occurring and three trace compounds. The hydrocarbon profile of *Camponotus rufifemur *consisted almost exclusively of methyl-branched alkenes of unusually high chain lengths (up to C_49_). This species occurred in two sympatric, chemically distinct varieties with almost no hydrocarbons in common. *Cr. modiglianii *discriminated between these two varieties. It only tolerated workers of the *Ca. rufifemur *variety it was associated with, but attacked the respective others. However, *Cr. modiglianii *did not distinguish its own *Ca. rufifemur *partner from allocolonial *Ca. rufifemur *workers of the same variety.

**Conclusion:**

We conclude that there is a mutual substance transfer between *Cr. modiglianii *and *Ca. rufifemur*. *Ca. rufifemur *actively or passively acquires cuticular steroids from its *Cr. modiglianii *partner, while the latter acquires at least two cuticular hydrocarbons from *Ca. rufifemur*. The cuticular substances of both species are highly unusual regarding both substance classes and chain lengths, which may cause the apparent inability of *Cr. modiglianii *to discriminate *Ca. rufifemur *nestmates from allocolonial *Ca. rufifemur *workers of the same chemical variety.

## Background

Associations across different animal taxa require specific adaptations on one or both sides. In particular, recognizing the partner species is a crucial task to any form of association, albeit in host-parasite associations only the latter might need to recognize the partner [1]. Nestmate recognition mechanisms in associating species must therefore go beyond the own species and include the partner species.

In ants, one of the closest and most intriguing interspecific associations is parabioses, where two ant species live together in a common nest. This phenomenon is found in several parts of the world, including Southeast Asia [2] and tropical South America [3]. Parabiotic ants have nestmates not only from their own colony, but also from a completely different species. Their nestmate recognition system therefore needs to include allospecific nestmates. In ants and other social hymenoptera, recognition is based on colony-specific chemical cues on the body surface that are perceived through olfactory or contact chemoreception [4,5]. Most of them are hydrocarbons [6-8]. Via allogrooming and trophallaxis, the individuals continually take up their nestmates' surface compounds into the postpharyngeal gland (PPG), where they are mixed and redistributed. Through this process, a colony-specific odour is created [5,9-11]. This colony-specific odour is learned by the colony members and represented as a neuronal template in the nervous system [12]. Nestmates are recognized by comparing the cuticular profile of the encountered individual to the neuronal template (phenotype matching), whereby a mismatch generally results in aggression [5].

Despite this complex nestmate recognition system, a considerable number of insect species manages to be accepted in Hymenoptera colonies, such as Lycaenid larvae, Staphylinidae, Ensifera, and Diptera [13-16] as well as social parasites, such as the parasitic bumblebee *Psithyrus *[17] and inquiline ant species [1,6]. In many of these associations, the parasite chemically resembles the host (chemical mimicry) [1,13-16,18,19]. Another possible mechanism to remain incognito is chemical insignificance [1]. Several social parasite species are – like callows – chemically insignificant, i.e. they do not possess an individual surface profile and are hence not recognized as foreign by their hosts [1,20,21]. Hydrocarbon profiles of very long chain lengths are difficult to perceive and hence may also promote chemical insignificance [22,23]. Still, numerous other social parasite species possess distinct profiles that do not resemble their hosts. Since these profiles neither show chemical mimicry nor insignificance, it has been supposed that the host species habituate to the parasites' profiles [24-26].

While the chemical mechanisms of tolerance between species have been studied in associations like social parasitism, little is known about parabiotic associations. It seems likely that parabiotic ants possess a nestmate recognition system that tolerates allospecific nestmates. In the present study we examined the relationship between interspecific tolerance and surface chemistry among the Southeast Asian parabiotic species *Crematogaster modiglianii *and *Camponotus rufifemur*. The two species tolerate ants from certain (but not all) foreign parabiotic nests but attack non-parabiotic ant species [2]. We discovered that two morphological varieties of *Ca. rufifemur *(the 'red' and the 'black' variety, see Methods) also differ in their chemical profiles. This enabled us to study two different levels of chemical similarity – within and between the two varieties. Our research questions were:

(1) Do parabiotic species possess cuticular substances different from related, non-parabiotic species?

(2) Is there evidence for chemical mimicry, i.e., chemical overlap between parabiotic partners?

(3) Do chemical differences *within *species account for differences in interspecific allocolonial tolerance?

## Results

### Cuticular substances: Hydrocarbons and other aliphatic components

**Figure 1:**
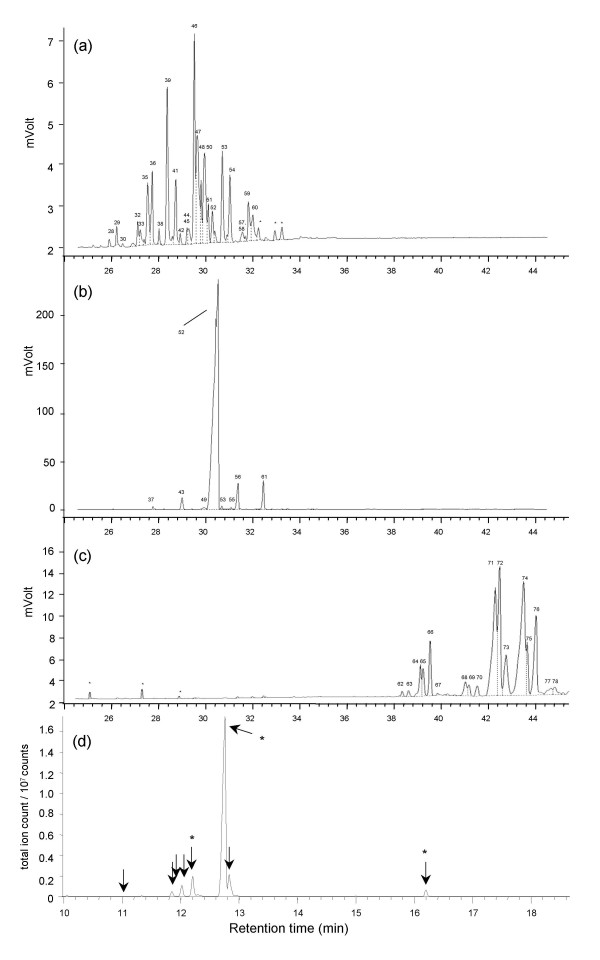
**Gas chromatograms of cuticular hydrocarbons of the parabiotic ant species**. (a) *Crematogaster modiglianii *B2, (b) red *Camponotus rufifemur *R2, (c) black *Camponotus rufifemur *B4. Graphs were acquired with a GC-FID. Only substances beyond a chain length of 34 are shown since shorter hydrocarbons make up less than 2% of the profile. Numbers refer to table 1. *unknown, irregularly occurring substance. (d) Typical chromatogram of the cuticular steroids of *Cr. modiglianii*, acquired with GC-MS. Arrows indicate the steroid compounds common to both *Cr. modiglianii *and *Ca. rufifemur*. Asterisks indicate the three steroids with highly similar mass spectra used for the second Mantel test. No other steroids were present in the colony shown.

The cuticular profile of both *Camponotus rufifemur *and *Crematogaster modiglianii *highly differed from other, non-parabiotic *Camponotus *and *Crematogaster *species [9,27-29]; unpublished data]. While there were only few aliphatic compounds with a chain length of C20-C33, both species possessed hydrocarbons of very high chain lengths (C35 up to C49, Figure 1) as well as steroids, which have not previously been detected on insect cuticles. The aliphatic profile of *Crematogaster modiglianii *consisted of hydrocarbons between C33 and C40. Beside n-alkanes and methyl-branched alkanes, more than 68% of its aliphatic cuticular compounds were unsaturated (Figure 1a, Tables 1, 2). Extracts of the body surface and postpharyngeal glands contained the same aliphatic substances in similar quantitative composition.

**Table 1:** Aliphatic cuticular substances found in *Crematogaster modiglianii *and the two varieties of *Camponotus rufifemur*

	**substance**	**substance class**	**retention index**	**red *Ca. rufifemur***	**black *Ca. rufifemur***	***Cr. modiglianii***
1	C21	n-alkane	21		0.26 ± 0.01%	
2	C23:1	n-alkene	22.75		0.46 ± 0.03%	
3	unknown	unknown	22.9		0.20 ± 0.01%	
4	C23	n-alkane	23		0.16 ± 0.01%	
5	C24:1	n-alkene	23.78		0.49 ± 0.06%	
6	Docosenal^+^	aldehyde	24.07		0.40 ± 0.09%	
7	Docosenal^+^	aldehyde	24.12		0.20 ± 0.16%	
8	unknown	unknown	24.35		0.23 ± 0.06%	
9	12-MeC24	branched alkane	24.37	0.06 ± 0.05%		
10	11-MeC24	branched alkane	24.39		0.45 ± 0.02%	
11	C25	n-alkane	25			
12	Tricosenal^+^	aldehyde	25.11		0.11 ± 0.09%	
13	unknown	unknown	25.69	0.05 ± 0.02%		
14	unknown	unknown	25.7		0.67 ± 0.17%	
15	C26	n-alkane	26	0.02 ± 0.01%		
16	Tetracosenal^+^	aldehyde	26.09		0.72 ± 0.17%	
17	unknown	unknown	26.37		0.21 ± 0.03%	
18	unknown	unknown	26.72		0.08 ± 0.02%	
19	C27	n-alkane	27	0.13 ± 0.11%	0.13 ± 0.01%	
20	Pentacosenal^+^	aldehyde	27.15		0.47 ± 0.39%	
21	unknown	unknown	27.71	0.09 ± 0.04%		
22	unknown	unknown	27.73		0.52 ± 0.08%	
23	C28	n-alkane	28	0.09 ± 0.06%	0.14 ± 0.01%	
24	C29	n-alkane	29	0.21 ± 0.17%	0.27 ± 0.02%	
25	C30	n-alkane	30	0.01 ± 0.01%	0.20 ± 0.03%	
26	C31	n-alkane	31	0.15 ± 0.11%		
27	C32	n-alkane	32		0.08 ± 0.03%	
28	C35:1	n-alkene	34.85		0.26 ± 0.09%	0.15 ± 0.04%
29	C35	n-alkane	35.05		0.15 ± 0.06%*	0.76 ± 0.11%
30	17-MeC35, 15-MeC35, 13-MeC35	branched alkane	35.31			0.88 ± 0.25%
31	3-MeC35	branched alkane	35.74			0.3 ± 0.13%
32	C37:2	n-alkadiene	36.42			2.31 ± 0.28%
33	C37:2	n-alkadiene	36.51			1.56 ± 0.12%
34	C37:2	n-alkadiene	36.64			0.45 ± 0.07%
35	C37-13-ene, C37-14-ene, C37-15-ene, C37-16-ene	n-alkene	36.72			5.43 ± 0.49%
36	C37-9-ene	n-alkene	36.86		0.48 ± 0.15%	4.53 ± 0.4%
37	25-MeC37-14-ene, 25-MeC37-16-ene^++^	branched alkene	36.96	0.44 ± 0.07%		
38	C37	n-alkane	37.05			0.52 ± 0.1%
39	19-MeC37, 17-MeC37, 15-MeC37, 13-MeC37, 11-MeC37	branched alkane	37.31			11.47 ± 0.28%
40	C38:2	n-alkadiene	37.45			0.18 ± 0.09%
41	11,27-DiMeC37, 11,25-DiMeC37	branched alkane	37.58			6.02 ± 0.33%
42	unknown	unknown	37.79			0.63 ± 0.1%
43	x(25,26,27)-MeC38-y(13,14,15,16)-ene^++§^	branched alkene	37.93	1.99 ± 0.11%		
44	C39:3	n-alkatriene	38.23			1.12 ± 0.13%
45	C39:3	n-alkatriene	38.3			1.46 ± 0.19%
46	C39:2	n-alkadiene	38.43			15.23 ± 0.76%
47	C39:2	n-alkadiene	38.53			13.7 ± 0.73%
48	C39-ene	n-alkene	38.73			3.66 ± 0.34%
49	unknown	unknown	38.79	0.55 ± 0.08%		
50	C39:1	n-alkene	38.79			7.62 ± 0.19%
51	C39:1	n-alkene	38.88			1.7 ± 0.08%
52	27-MeC39-14-ene, 27-MeC39-16-ene	branched alkene	39.02	88.66 ± 0.53%		3.15 ± 1.18%
53	19-MeC39, 17-MeC39, 15-MeC39, 13-MeC39, 11-MeC39	branched alkane	39.29	0.52 ± 0.24% (only 13-MeC39)		4.51 ± 0.2%
54	11,21-DiMeC39, 11,23-DiMeC39, 11,27-DiMeC39, 11,29-DimeC39	branched alkane	39.54			4.84 ± 0.52%
55	unknown	unknown	39.76	0.22 ± 0.01%		
56	27-MeC40-14-ene, 27-MeC40-15-ene, 27-MeC40-16-ene^++^	branched alkene	39.97	3.41 ± 0.09%		
57	unknown	unknown	40.17			1.04 ± 0.18%
58	C40:3	n-alkatriene	40.35			0.36 ± 0.08%
59	C40:2	n-alkadiene	40.42			3.39 ± 0.24%
60	C40:2	n-alkadiene	40.57			3.01 ± 0.29%
61	x(27,29)-MeC41-y(14,16,18)-ene^++§^	branched alkene	40.94	3.35 ± 0.4%		
62	unknown	unknown	44.54		0.65 ± 0.12%	
63	unknown	unknown	44.68		0.45 ± 0.03%	
64	unknown	unknown	44.96		3.34 ± 0.19%	
65	C45:1	n-alkene	45.05		3.01 ± 0.04%	
66	36-MeC45:1	branched alkene	45.18		4.17 ± 0.06%	
67	unknown	unknown	45.49		1.09 ± 0.4%	
68	unknown	unknown	45.89		2.10 ± 0.16%	
69	unknown	unknown	45.97		0.98 ± 0.06%	
70	unknown	unknown	46.11		1.07 ± 0.06%	
71	C47:2	n-alkadiene	46.41		15.11 ± 0.52%	
72	C47:2	n-alkadiene	46.67		8.72 ± 0.37%	
73	C47:1	n-alkene	46.74		4.43 ± 0.18%	
74	C48:1	n-alkene	46.88		22.95 ± 0.96%	
75	C48:1	n-alkene	47.10		4.10 ± 0.07%	
76	38-MeC47:1	branched alkene	47.16		9.20 ± 0.49%	
77	unknown	unknown	47.42		1.49 ± 0.12%	
78	unknown	unknown	47.46		1.29 ± 0.11%	
79	unknown	unknown	47.81		1.91 ± 0.13%	
80	unknown	unknown	48.01		0.54 ± 0.07%	
81	C49:2	n-alkadiene	48.35		2.55 ± 1.7%	
82	C49:2	n-alkadiene	48.45		2.40 ± 1.6%	
83	C49:1	n-alkene	48.59		1.25 ± 0.11%	

Relative peak areas (mean and standard error) for *Ca. rufifemur *and *Cr. modiglianii *are given based on FID data from n = 6 (red *Ca. rufifemur*), 3 (black *Ca. rufifemur*), and 8 (*Cr. modiglianii*) colonies. *found in less than 50% of the samples, ^+^tentatively identified, ^++^position of double bond tentative, § number of substances and their exact structure could not be further determined. Retention indices beyond 44 are extrapolated.

**Table 2:** Relative quantities of the different aliphatic substance classes in *Cr. modiglianii *and *Ca. rufifemur*.

**substance class**	**red *Ca. rufifemur***	**black *Ca. rufifemur***	***Cr. modiglianii***
n-alkane	0.64 ± 0.41%	1.25 ± 0.18%	1.29 ± 0.16%
n-alkene	0 ± 0%	37.44 ± 0.94%	23.06 ± 1.03%
n-alkadiene	0 ± 0%	28.77 ± 2.41%	39.83 ± 1.09%
n-alkatriene	0 ± 0%	0 ± 0%	2.8 ± 0.16%
branched alkane	0.58 ± 0.26%	0.45 ± 0.02%	28.07 ± 0.85%
branched alkene	98.1 ± 0.35%	13.37 ± 0.44%	3.15 ± 1.18%
aldehyde	0 ± 0%	1.9 ± 0.78%	0 ± 0%
unknown	0.91 ± 0.11%	16.82 ± 0.42%	1.77 ± 0.28%

Mean and standard error are given, based on FID data from n = 6 (red *Ca. rufifemur*), 3 (black *Ca. rufifemur*), and 8 (*Cr. modiglianii*) colonies.

The *Camponotus rufifemur *surface profile mainly contained compounds beyond C_38_, beside traces of lighter components. The two morphological varieties exhibit almost completely different surface profiles. The only substances in common were trace n-alkanes between C27 and C30 and C37-9-ene (Table 1). The red variety exhibited a highly unusual cuticular profile, 98% of the hydrocarbon quantities being methyl-branched alkenes. The main compounds, 27-MeC39-14-ene and 27-MeC39-16-ene, accounted for 88.7% of the total hydrocarbons. The other different methyl-branched alkenes were similar in respect to the positions of the methyl group and the double bond (Table 3). Chain lengths ranged from C38 to C41, with trace compounds between C24 and C37 (Figure 1b, Tables 1,2).

**Table 3:** Diagnostic ions of the methyl-branched alkenes in the red *Ca. rufifemur *variety.

**substance No.**	**Substance**	**diagnostic ions from hydration**	**diagnostic ions from DMDS derivatization**	**inferred double bond position**
37	25-MeC37-ene	196, 365	243*, 271, 355, 383*	14*, 16 or 21, 23*
43	25-MeC38-ene, 26-MeC37-ene, 27-MeC38-ene	182, 196, 210, 364, 379, 393	229, 243, 257, 271, 369, 383, 397, 411	13, 14, 15, 16 or 22, 23, 24, 25
52	27-Methyl-C39-ene	196, 393	243, 271, 383, 411	14**, 16** or 23, 25
56	27-Methyl-C40-ene	210, 393	243*, 257, 271, 397, 411, 425*	14*, 15, 16 or 24, 25, 26*
61	27-MeC41-ene, 29-MeC41-ene	196, 224, 392, 421	243, 271*, 299, 383, 411*, 439	14, 16*, 18 or 23, 25*, 27

*diagnostic ion/molecule with respective double bond position at least twice as abundant as remaining ions/molecules; ** position of double bond was confirmed at the positions 14 and 16 via cleavage after ozonisation

The profile of the black *Ca. rufifemur *variety consisted of even larger molecules, with 92.8% of the surface compounds between C44 and C49 (Table 1). At least 80% of the compounds were unsaturated (Table 2). Methyl-branched alkenes were also present, albeit not as abundant as in the red *Ca. rufifemur *variety. Minor compounds included n-alkanes, methyl-branched alkanes and aldehydes (Table 2). In both *Ca. rufifemur *varieties, PPG and surface extracts contained the same aliphatic compounds in similar relative quantities.

### Cuticular substances: Steroids

Besides aliphatic compounds, the surface profile of both ant species contained up to 24 components with a basic steroid structure (as inferred from mass spectra and diagnostic ions). Their mass spectra indicate a close chemical interrelatedness of the compounds. Due to the high substance quantities necessary for NMR analysis, their spatial molecular structure has not yet been resolved but is under investigation. *Crematogaster modiglianii *possessed high amounts of steroids on the body surface (2.59 ± 0.58 μg/worker, n = 11 colonies, mean and SE) which by far exceeded the hydrocarbons (0.48 ± 0.05 μg/worker, n = 11 colonies, mean and SE). In contrast, postpharyngeal gland extracts only contained minor amounts of steroids but high quantities of hydrocarbons. High steroid amounts of the same quantitative composition were also found in the Dufour gland, in separate alitrunk and gaster cuticular extracts and, albeit in lower amounts, in head cuticular extracts. They also occurred in cuticular extractions of living ants with SPME fibres, thus confirming that their presence in hexane extracts was not an artefact of concomitantly extracted glands. Altogether, *Cr. modiglianii *extracts contained 24 different steroid components with an abundance higher than 0.1% in at least one colony (percent of total steroid abundance). Their retention indices ranged between 20.38 and 25.77. Six of the 24 steroids were found in all *Cr. modiglianii *colonies in similar relative compositions. An additional eleven steroids were abundant in certain colonies but absent in others. The remaining seven steroids were irregularly found and never occurred in relative abundances higher than 1% (percent of total steroid abundance). *Camponotus rufifemur *extracts (both varieties) contained up to eight different steroids, all of which also occurred in *Cr. modiglianii*. The absolute steroid quantities in *Ca. rufifemur *were lower than the hydrocarbon quantities (black variety: 0.66 ± 0.22 μg steroids/worker and 1.79 ± 0.29 μg hydrocarbons/worker, n = 3 colonies; red variety: 0.41 ± 0.14 μg steroids/worker and 9.71 ± 3.79 μg hydrocarbons/worker, n = 4 colonies, mean and SE given).

### Chemical overlap among the parabiotic species

**Figure 2:**
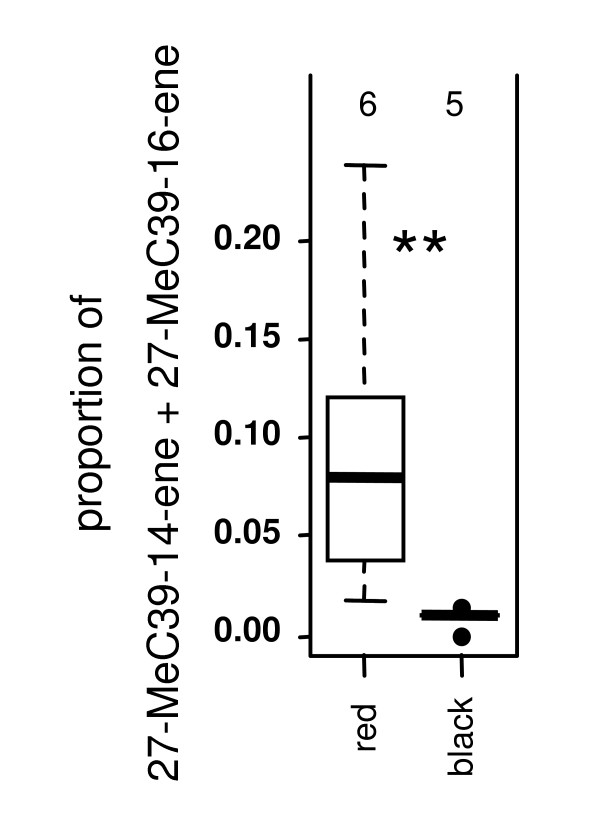
**Relative abundance of 27-MeC39-14-ene and 27-MeC39-16-ene in *Cr. modiglianii *workers living with the red vs. *Cr. modiglianii *workers living with the black *Ca. rufifemur *variety**. Median, quartiles, range, and outliers (i.e. all data points deviating from the box by more than 1.5 times the interquartile range) are shown in the present and the following figures. The number of analyzed colonies is given above each plot. ** highly significant (p = 0.0043) according to U test.

Six hydrocarbons were shared between both parabiotic species. The red *Ca. rufifemur *variety shared three hydrocarbons with *Cr. modiglianii*. These were the two methyl-branched alkenes, 27-MeC39-14-ene and 27-MeC39-16-ene, which are the main constituents of the red *Ca. rufifemur *surface profile, and its saturated derivative, 13-MeC39 (Table 1). All three are absent in the black *Ca. rufifemur *variety. *Cr. modiglianii *colonies living with the red *Ca. rufifemur *variety (henceforth, 'red' *Cr. modiglianii*) exhibited significantly more 27-MeC39-14-ene and 27-MeC39-16-ene than those associated with the black variety (henceforth, 'black' *Cr. modiglianii*) (Mann-Whitney W = 30, p = 0.0043; N_1 _= 5, N_2 _= 6 colonies, Figure 2). The quantities of 13-MeC39 were not compared since they could not be separated from other methyl-branched C39 alkanes in *Cr. modiglianii *(Table 1). Traces of three other hydrocarbons common in *Cr. modiglianii *were detected in the black *Ca. rufifemur *variety (C35:1, C35, C37-9-ene, Table 1). Albeit the associated *Cr. modiglianii *possessed slightly more C37-9-ene than those living with the red variety, no significant differences were found.

Eight of the steroids common in *Cr. modiglianii *were also frequently found in *Ca. rufifemur *(inclusion criterion: median abundance > 0% in 11 colonies of both species; Figure 1d). Their relative abundances varied between parabiotic nests but were significantly correlated among the two species within a nest (Mantel test: r = 0.49, p = 0.041, N = 11; Bray-Curtis distances: 0.13 ± 0.08 (*Ca. rufifemur*), 0.43 ± 0.31 (*Cr. modiglianii*); mean and s.d.). A second Mantel test considered only three steroids with very similar mass spectra, which were present in all extracts (retention indices: 21.92, 22.24, 24.47; marked with asterisks in Figure 1d). This test yielded a highly significant correlation of steroid abundance among the two species of each parabiotic nest (r = 0.620, p < 0.001, N = 11; Bray-Curtis distances: 0.06 ± 0.04 (*Ca. rufifemur*), 0.13 ± 0.07 (*Cr. modiglianii*)).

### Differences in allocolonial tolerance

**Figure 3:**
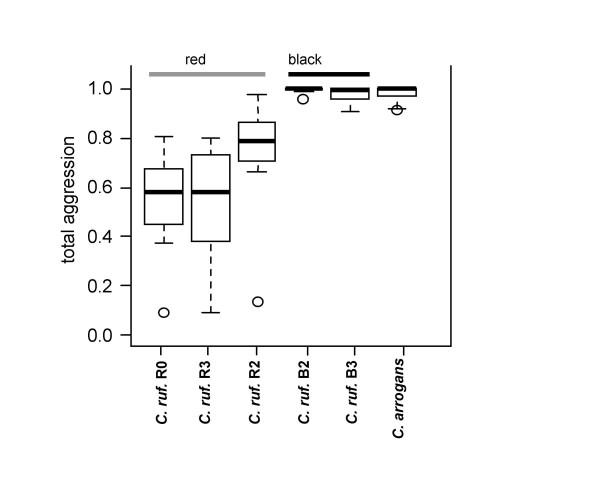
**Total aggression of *Crematogaster modiglianii *(colony R0) against different *Camponotus *colonies and species**. Data are given as proportions in relation to the total number of interactions. Each plot represents 10 replicates.

Chemical differences between the two *Ca. rufifemur *varieties accounted for much of the variance in interspecific confrontations. In general, *Cr. modiglianii *workers tolerated only allocolonial *Ca. rufifemur *workers of the variety they were associated with. The focal *Crematogaster modiglianii *colony, which lived together with the red *Ca. rufifemur *variety, showed high aggression towards dead workers of the black *Ca. rufifemur *variety but not towards those of the red one (Figure 3). The generalized linear model (GLM) for total aggression explained 65.6% of the total deviance and yielded a highly significant effect of the *Camponotus *variety (58.7% explained deviance, Table 4). The remaining deviance could in part be attributed to differences between *Camponotus *colonies (p = 0.04), whereas the difference between intracolonial and allocolonial *Camponotus *was not significant (p = 0.12, Table 4). The non-parabiotic *Camponotus (Tanaemyrmex) arrogans *was attacked to a similar degree as the black *Ca. rufifemur *variety (Figure 3). When the analysis focused on the proportion of strong aggression only, the results were similar, with slightly stronger effects. *Cr. modiglianii *very rarely climbed onto the *Ca. rufifemur *bodies in this experimental series ('mounting behaviour').

**Table 4:** GLM for total aggression of *Cr. modiglianii *towards dead *Ca. rufifemur *workers from different colonies.

**Parameter**	**Deviance**	***df***	***F***	***P***
*Ca. rufifemur *variety	735.3	1	74.16	< 0.0001
*Ca. rufifemur *colony	62.8	2	3.45	0.040
intra-/allocolonial	24.7	1	2.53	0.12
residual error	430.0	46		
total	1252.9	50		

Data from behavioural experiments with a *Cr. modiglianii *laboratory colony. '*Ca. rufifemur *colony' is nested within '*Ca. rufifemur *variety'.

**Figure 4:**
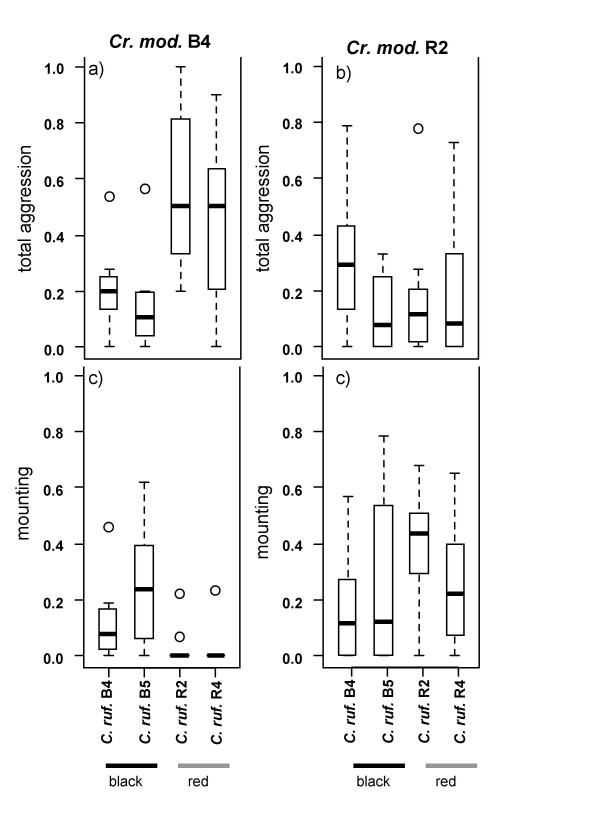
**Total aggression (a, b) and mounting behaviour (c, d) of *Cr. modiglianii *towards dead *Ca. rufifemur *from different colonies in arena assays**. Data are given as proportions in relation to the total number of interactions. Each plot represents 10–13 replicates. (a), (c) *Cr. Modiglianii *B4, (b), (d) *Cr. modiglianii *R2.

In the arena confrontations, *Cr. modiglianii *was significantly more aggressive towards *Ca. rufifemur *from the respective other variety. The parameter 'within/across variety' explained 18.2% of the total deviance, followed by 'variety combination' (13.0% explained deviance), while 'intra-/allocolonial' did not explain a significant part of the deviance (Table 5). *Cr. modiglianii *workers frequently climbed on *Ca. rufifemur *bodies and walked around on them for up to one minute. This 'mounting behaviour' represented on average 18.6% of all interactions (Figure 4). The workers (especially in one of the two colonies) mounted *Ca. rufifemur *workers of their 'own' variety in significantly higher proportions (GLM for both colonies: F_df  = 1 _= 6.85, p = 0.011) but did not otherwise differentiate between intracolonial and allocolonial *Ca. rufifemur *workers (F_df  = 1 _= 0.14, p = 0.71).

**Table 5:** GLM for total aggression of *Cr. modiglianii *towards dead *Ca. rufifemur *from different colonies.

**Parameter**	**Deviance**	***df***	***F***	***P***
within/across varieties	107.8	1	20.64	< 0.0001
variety combination	77.3	2	8.19	0.00056
colony combination	37.0	3	2.76	0.048
intra-/allocolonial	0.2	1	0.05	0.83
residual error	370.6	80		
total	592.9	87		

Data from arena confrontations with *Cr. modiglianii*. 'Variety combination' ist nested within the parameter 'within/across varieties'. 'Colony combination' is nested within 'variety combination'. Due to this nested structure, no interactions between the variables occur.

**Figure 5:**
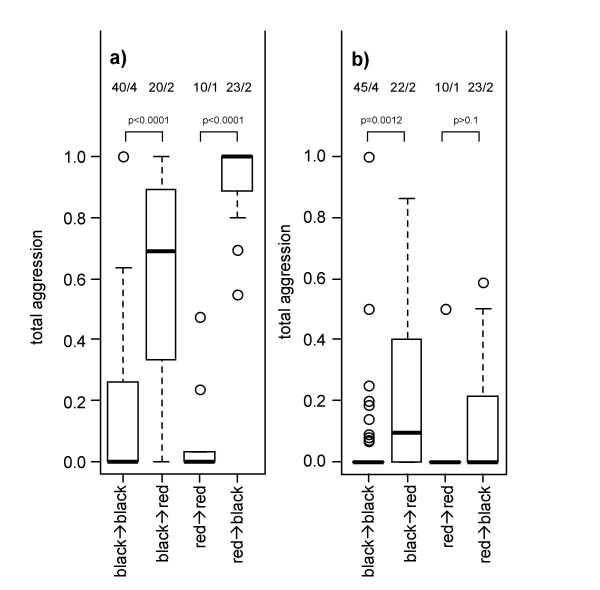
**Total aggression in allocolonial confrontations between parabiotic partners assays, pooled for variety combinations**. Data are from [2], given as proportions in relation to the total number of interactions. The numbers above each plot indicate the overall number of replicates and the number of colony combinations tested. P values are given according to GLMs with binomial error distribution. (a) *Cr. modiglianii *towards *Ca. rufifemur*, (b) *Ca. rufifemur *towards *Cr. modiglianii*.

In order to examine whether the differentiation between the colour varieties occurred in colonies *in situ *as well, we re-analyzed previous behavioural experiments reported in [2]. Allocolonial aggression of *Cr. modiglianii *towards *Ca. rufifemur *was highly variable in this dataset, and we confirmed a high impact of the two chemical varieties on allocolonial aggression. The variable 'within/across varieties' (colonies A and B: black variety, colony C: red variety) explained 60.1% of the total variance of the data and was a clearly more powerful predictor than the differentiation between intra- vs. allocolonial combination (0.03% deviance explained, Table 6). 'Red' *Cr. modiglianii *colonies only attacked black *Ca. rufifemur *intruders and vice versa (Figure 5a). The highly significant impact of 'variety combination' (Table 6), however, showed that red *Cr. modiglianii *was more aggressive towards black *Ca. rufifemur *than black *Cr. modiglianii *towards red *Ca. rufifemur*.

**Table 6:** GLM for total aggression in interspecific live confrontations

	***Cr. modiglianii → Ca. rufifemur***				***Ca. rufifemur → Cr. modiglianii***			
	**Deviance**	***df***	***F***	***P***	**Deviance**	***df***	***F***	***P***
within/across varieties	674.3	1	124.56	< 0.0001	45.2	1	13.09	0.00047
variety combination	79.4	2	9.19	0.0002	5.6	2	0.81	0.45
intra-/allocolonial	0.3	1	0.07	0.79	0.0	1	0.00	0.99
colony combination	18.0	4	1.01	0.41	12.4	4	0.99	0.42
No. *Camponotus*	1.4	1	0.32	0.57	0.0	1	0.00	0.96
No. *Crematogaster*	2.1	1	0.47	0.49	5.2	1	1.73	0.19
residual error	346.0	82			260.5	89		
total	1121.5	92			328.9	99		

Data from [2]. 'Colony combination' is nested within 'variety combination'. There are no interactions between the variables due to their nested structure.

In confrontations of *Ca. rufifemur *towards allocolonial *Cr. modiglianii*, Menzel et al. [2] had found low levels of aggression albeit they were higher than against intracolonial *Cr. modiglianii*. Similar to above, *Ca. rufifemur *workers were more aggressive towards *Cr. modiglianii *from the respective other variety (Figure 5b, Table 6).

## Discussion

### Unusual features of the cuticular profiles in parabiotic ants

To our knowledge, steroids have not been found in surface extracts of other ant species up to now, and to our knowledge have been found on insect cuticles only in one halictid bee [30]. However, various *Crematogaster *species are known to have highly efficient poisons [31,32]. The genus *Crematogaster *has evolved a peculiar system of venom production which involves a cooperation of Dufour and poison gland. In several species the venom consists of precursors from the Dufour gland which are derivatized by enzymes from the poison gland [33,34]. *Crematogaster *poisons – from Dufour and poison glands, but also from hypertrophied metapleural glands – belong to such different chemical classes as cyclohexan derivatives, crematofuranes (cembranoid diterpenes), coumarin derivatives, alkylphenols, alkylresorcinols, salicylic acids, resorcylic acids, and polyfunctionalized long-chain derivatives [33,35-38]. Since extracts of *Cr. modiglianii *Dufour glands contained the same steroid composition as the body surface (but no other compounds), they are probably produced in this gland and then distributed onto the body surface. In *Cr. modiglianii*, steroid synthesis did not depend on biosynthetic precursors acquired from food. In two colonies kept in the laboratory for 15 and 6 months, respectively, the steroid profile did not change despite of an artificial diet of cockroaches, honey solution and Bhatkar diet (F.M. pers. obs.). Moreover, in one forest colony, the steroid profile remained relatively constant over three years, corroborating that the steroid composition is rather genetically determined than dependent on environmental factors.

It is notable that 98% of the entire hydrocarbon profile of the red *Ca. rufifemur *(and ≥ 13% of the black *Ca. rufifemur *hydrocarbon profile) were methyl-branched alkenes. This substance class seems to be generally very rare in insects and has been detected only in several Diptera and one Noctuid moth as pheromones [39-41]. Among ants, they have been found in traces in the ponerine ant *Pachycondyla villosa *and in two *Leptothorax *species [42,43], but in higher abundances only in *Nothomyrmecia macrops *surface profiles, which is probably the most primitive existent ant species [44]. That they make up almost the entire hydrocarbon profile is therefore highly unusual. Another unusual feature in both parabiotic species is the high hydrocarbon chain lengths. Although common in this study (Table 1), hydrocarbons beyond C_37 _have not been found in non-parabiotic *Camponotus *and *Crematogaster *species [9,27,28]; unpublished data. Other studies report small concentrations of heavier hydrocarbons in other ant genera, but always accompanied by high amounts of lighter ones [45,46]. It is possible that extremely long-chain hydrocarbons are difficult to perceive by receptors and thus promote interspecific tolerance [23,47]. In one case, we observed that a non-parabiotic *Cr. modiglianii *colony was initially very aggressive against (black) *Ca. rufifemur *workers but treated them amicably (and had hence become habituated) after less than 24 h of exposure. Unsaturation in these long-chain hydrocarbons might be necessary to maintain a minimum fluidity of the cuticular profile [47].

### Chemical overlap among parabiotic partners

Given the high allocolonial tolerance between parabiotic partners, the hydrocarbon overlap of the two species is surprisingly small. While the red *Ca. rufifemur *variety shared two compounds with its partner, the black variety only shared three trace compounds with *Cr. modiglianii *but otherwise possessed a completely different hydrocarbon profile. We tentatively suppose that *Cr. modiglianii *acquires 27-MeC39-14-ene 27-MeC39-16-ene from its red *Ca. rufifemur *partner although *Ca. rufifemur *generally tolerates *Cr. modiglianii *workers, including those lacking these substances [2]. In a *Cr. modiglianii *colony kept in the laboratory without its previous red *Camponotus *partner, the compound disappeared from the profile after eight months of separation (F.M. pers. obs.). It is possible that the other hydrocarbons of the red *Ca. rufifemur *are acquired by *Cr. modiglianii *as well but remain beyond detectability due to their low abundances. The hydrocarbons of the black *Ca. rufifemur*, in contrast, were never found on *Cr. modiglianii *surface extracts. This is probably due to their high chain lengths, which makes the cuticular profile more solid and do not allow chemical transfer [47]. In the light of the low overall hydrocarbon overlap among the two parabiotic ant species, chemical camouflage, a mechanism often found in social parasites [13-15], must be dismissed as an explanation for mutual tolerance. However, the existence of only few substances common to both species might be a sufficient signal for tolerating the partner [48].

The steroid components, in contrast, showed high congruence among both species. We found that the relative composition of eight steroid compounds differs between colonies but is very similar among the two species of a parabiotic nest. Since it is highly improbable that *Ca. rufifemur *is able to synthetically copy the steroid profile of each respective partner colony, this result suggests that *Ca. rufifemur *acquires steroids from *Cr. modiglianii*. Notably, only a certain set of steroids is transferred to *Camponotus*, while others, despite of high abundance in *Cr. modiglianii*, were almost or completely absent from the *Ca. rufifemur *profile.

### Possible transfer mechanisms

Two mechanisms seem possible for the observed transfer of chemical cues, namely trophallaxis and direct physical contact. Via trophallaxis, individual ants exchange not only food but also the PPG content, i.e. hydrocarbons relevant for nestmate recognition [49]. The PPG of *Cr. modiglianii *indeed contained steroids, albeit in much lower concentrations than on the body surface, thus making trophallaxis a possible pathway for chemical transfer. Interspecific trophallaxis has been observed between the two parabiotic species (F.M. and A. Endler, pers. obs.) and also shown via stained food only fed to *Cr. modiglianii *(F.M., pers. obs.).

Another possible transfer mechanism is direct physical contact. We frequently observed that *Cr. modiglianii *climbed on living or dead *Ca. rufifemur *individuals (workers and alates). The latter sometimes tried to shake them off but did not show aggression. Though almost never observed in the field, this 'mounting behaviour' could be easily induced in the laboratory by keeping the two species separate for one or two days. Mounting may therefore represent another possible mechanism for transfer of surface chemicals.

### Partner recognition is not colony-specific

The red and the black variety of *Camponotus rufifemur *are chemically distinct and – apart from trace compounds – do not share any hydrocarbons. The two dominant surface components of the red variety (substance #52, Table 1) are present in *Crematogaster modiglianii *colonies associated with this *Ca. rufifemur *variety but almost completely absent from those living with the black variety. Their abundance thus allows separating 'red' from 'black' *Cr. modiglianii *albeit the remaining surface profile is similar. The existence of two chemical *Ca. rufifemur *varieties accounts for most of the aggression variance in allocolonial encounters between the two species. *Cr. modiglianii *usually tolerated living or dead *Ca. rufifemur *workers of the same variety as their parabiotic partner but fiercely attacked those of the respective other variety (Figures 3, 4, 5, Tables 4, 5, 6). An analogous pattern was found in *Ca. rufifemur*. Despite of generally low aggression levels, black *Ca. rufifemur *workers were significantly more aggressive towards 'red' *Cr. modiglianii *workers than towards allocolonial 'black' *Cr. modiglianii *(Figure 5b). However, we did not detect a corresponding difference in the red *Ca. rufifemur*.

While much of the interspecific aggression can be explained by chemical differences, however, the low interspecific aggression *within *chemical varieties is still surprising. Rather than recognizing heterospecific nestmates, the two species seemingly recognize only the chemical variety of their partner and do not discriminate within these varieties. Nestmate recognition rather depends on volatile substances than on substances only perceivable through antennal contact [50]. Due to their low volatility [47], very long-chain hydrocarbons are less detectable than short-chain molecules. Thus, olfactory receptors may additionally absorb traces of lighter hydrocarbons, thereby blurring inter-colony profile differences and hampering inter-colony discrimination [23]. The role of the steroids in the nestmate discrimination process is still unclear and under investigation.

The high interspecific tolerance strongly contrasts with the South American parabioses of *Crematogaster limata *and the ponerine ant *Odontomachus mayi*, where the ants never tolerated heterospecific workers from foreign parabioses [3]. In these associations, very low chemical overlap was found (no substance data given), suggesting that both species habituated to each other's colony-specific profiles. The associated Chilean species *Camponotus morosus *and *Solenopsis gayi *also showed distinct hydrocarbon profiles [51]. In contrast to non-associated colonies, however, associated *C. morosus *had acquired small amounts of the *S. gayi *hydrocarbons. In both of these species, only individuals from associated colonies were tolerant towards allocolonial allospecifics [51], indicating that the acquisition of allospecific hydrocarbons promoted mutual tolerance.

## Conclusion

In this study we document the cuticular chemistry of the parabiotically associated ant species *Camponotus rufifemur *and *Crematogaster modiglianii*. In contrast to neotropical parabioses, these ant species did not show heterospecific nestmate recognition. In our experiments, *Cr. modiglianii *did not discriminate its partner *Ca. rufifemur *colony from other *Ca. rufifemur *colonies of the same chemical variety (nor vice versa). Rather, *Cr. modiglianii *distinguished only between the two *Ca. rufifemur *varieties, accepting the familiar one but attacking the respective other. This reduced discrimination of heterospecific nestmates may be caused by two unusual properties of the cuticular surface: Transfer of *Ca. rufifemur *hydrocarbons to the *Cr. modiglianii *profile (in one of the *Ca. rufifemur *varieties only), and the generally high chain hydrocarbon lengths in the two parabiotic species. As hypothesized elsewhere [23], extremely long-chain hydrocarbons may be difficult to perceive by receptors and hence promote chemical insignificance (sensu [1]). It is currently investigated whether the cuticular steroids unique to these species play a role in nestmate or partner recognition.

## Methods

### Study site and ants

The studies were conducted at Danum Valley Conservation Area from September to November in the years 2004 and 2007. Danum Valley represents one of the major remaining patches of tropical lowland rainforest in Sabah (Malaysian Borneo). The site has a typical equatorial rainforest climate with a mean annual temperature of 26.9°C and a yearly rainfall of 2700 mm. We studied parabiotic associations of *Camponotus (Myrmotarsus) rufifemur *Emery 1900 and *Crematogaster (Paracrema) modiglianii *Emery 1900. Their nests are commonly found in hollow, living tree trunks in the rainforest. Extracts of one parabiotic nest from the Kuala Belalong Field Studies Center (Brunei) were analyzed in addition.

*Camponotus rufifemur *occurs in two sympatric morphological varieties that have not previously been described (although Emery [52] notes that specimen from Sarawak are darker in colour than those from Sumatra). While one variety (henceforth, 'red' variety) has a reddish alitrunk and light red-brown legs, the other one (henceforth, 'black' variety) possesses a black alitrunk and dark red-brown legs. The area between the frontal carinae of soldiers is dull in the red but shining in the black variety. Although the ratio head width/scape length (in frontal view) tends to be higher in the large soldier caste of the black variety than in that of the red variety, no significant morphometric differences were found. In the following, we will refer to the varieties as 'red' and 'black' *Ca. rufifemur*. In order to allow a differentiation, the respective associated *Cr. modiglianii *will be called 'red' and 'black' *Cr. modiglianii *although we did not find morphological distinctions within this species. Voucher specimen of *Cr. modiglianii *and both *Ca. rufifemur *varieties are deposited at the Department of Zoology III, University of Würzburg and at the Forest Resarch Center in Sepilok, Sabah (Malaysia).

### Preparation of extracts

Extracts were prepared from both body surface and postpharyngeal glands (PPGs). For body rinses, 10 to 90 ants were killed by freezing and immersed in hexane for ten minutes. Extracts from single individuals contained quantities too low for reliable substance identification. Eleven parabiotic nests were sampled with one to eight (mean: 3.5) replicates per colony and species (ten from Danum Valley, one from Kuala Belalong). PPG extracts were obtained from three to four freshly dissected PPGs per sample dissolved in hexane. Octadecane (*n*-C_18_) was used as internal standard in most samples. Cuticular substances were additionally obtained from living *Cr. modiglianii *workers brought into the laboratory in Würzburg with solid-phase microextraction (SPME). A SPME fibre (Supelco) coated with a 100 μm polydimethylsiloxan film was rubbed on the ant for 3 min and then directly injected into a ThermoQuest Trace GC.

### Chemical analysis

Substances were identified by coupled capillary gas chromatography-mass spectrometry (GC-MS) with a Hewlett Packard 6890 series gas chromatograph coupled to a HP 5973 Mass Selective Detector. The GC was equipped with a J&W Scientific DB-5 fused silica capillary column (30 m × 0.25 mm ID; df = 0.25 μm). Temperature was kept at 60°C for 2 min then increased by 60°C/min up to 200°C and subsequently by 4°C/min to 320°C, where it remained constant for 10 min. Helium was used as carrier gas with a constant flow of 1 ml/min. A split/splitless injector was installed at 250°C in the splitless mode for 30 s. The electron impact mass spectra (EI-MS) were recorded with an ionisation voltage of 70 eV, a source temperature of 230°C and an interface temperature of 325°C. For analysis of hydrocarbons beyond C41, we used a DB-1 HT column (30 m × 0.25 mm ID; df = 0,25 μm). Temperature was raised from 60°C by 5°C/min up to 350°C and then kept constant for 10 min. The interface had a temperature of 350°C. All other settings were as above. The software MSD ChemStation (Version A.03.00) for Windows was used for data acquisition. We restricted the analyses to substances with a retention time beyond that of C19 since compounds with shorter chain length are likely to be too volatile to be relevant for nestmate recognition [5,6]. Substances present in less than 50% of the samples are given in Table 1 (marked with *) but were disregarded from further analysis.

For quantification of steroid-like compounds and aliphatics shorter than C_33_, we used ion counts from the GC-MS data and analysed both substance classes separately. Heavier hydrocarbons (beyond C33) were quantified using a high-resolution ThermoQuest Trace GC-FID with H_2 _as carrier gas in order to achieve a better separation of the substances. We used a nonpolar capillary column [DB1 (J&W Scientific, Folsom, CA), 20 m × 0.18 mm, 0.18 μm film thickness] and the first temperature program given above (split closed for 30 s for extracts and for 2 min when using SPME fibers). The split/splitless injector port was kept at 260°C and the flame ionization detector (FID) at 340°C. Peak areas were computed with Chrom-Card 1.19 (CE Instruments, Milan, Italy). Mean absolute substance quantities were estimated by comparing substance peak areas with that of the internal standard (acquired with GC-FID) and dividing by the number of extracted individuals.

Profile similarities between the two partner species were analyzed for eleven parabiotic nests (including one from Kuala Belalong Field Studies Center). The average proportions of the steroid components per colony and species were calculated. The distances between colonies were calculated for each species separately using Bray-Curtis index of similarity and then compared between species using a Mantel test (1000 permutations).

### Identification of cuticular hydrocarbons

Alkanes, methyl-branched alkanes and alkenes were characterized using diagnostic ions and retention indices calculated using Kovats' method [53]. Unsaturated methyl-branched hydrocarbons were hydrated under a H_2 _atmosphere using Palladium on activated carbon as catalyst to determine the position of the methyl group. The position of the double bond in methyl-branched and n-alkenes was determined using DMDS derivatization following [54]. For methyl-branched alkenes, DMDS derivatization was insufficient for substance characterization since the position of the double bond relative to the methyl group remained unresolved and left two possible structures. Therefore, we cleaved the molecules in two parts at the position of the double bond via ozonisation. We diluted the sample in approx. 3 ml hexane, applied a constant flow of O_3 _(300 mg/h) for ten minutes from a glass pipette (EO3G Ozone Generator, Easelec Technology Inc.) and directly injected the sample into the GC-MS. Ozonisation succeeded for substance 52 but not for the substances 37, 43, 56, and 61 (surface compounds of the red *Ca. rufifemur*, Table 1). However, it is highly probable that all methyl-branched alkenes are produced via the same biosynthetic pathway. We therefore tentatively inferred the position of the double bond from the structure of substances 52 (Table 1) and possibilities left from the DMDS results, which had succeeded for all of the above substances. Double bond positions in alkenes with chain lengths higher than C41 as well as in dienes and trienes could not be determined due to their low abundance and/or their high chain length, which resulted in derivatives which could not be detected using GC-MS. Aldehydes were identified by comparing their mass spectra to a commercial library (Wiley 275) and therefore remain tentative.

For the substances of the black *Ca. rufifemur *profile beyond C44, retention indices were calculated based on the retention times of an n-alkane standard (C21 to C40), C47 and C49, and therefore remain preliminary. These substances were identified based on mass spectra and hydrated samples. Unsaturation was further confirmed via fractionation using a SiOH column treated with AgNO_3_. However, their characterization remains preliminary since the DMDS derivatized substances could not be detected using GC-MS.

### Behavioural experiments

We studied the reaction of *Crematogaster modiglianii *towards dead *Ca. rufifemur *workers from different colonies in Borneo. The reverse situation (*Ca. rufifemur *towards *Cr. modiglianii*) was not studied in this paper since *Ca. rufifemur *shows little discrimination between different *Cr. modiglianii *workers [2]. A *Cr. modiglianii *colony (R0) had been collected in the forest circa one week prior to the experiments and was kept together with its red *Ca. rufifemur *partner in its original nest (a small tree trunk) in an open plastic box. The dead ants were placed onto the nest trunk with forceps such that several ants could interact with it simultaneously. During three minutes, each observed interaction was classified as peaceful (antennating), weakly (open mandibles) or strongly aggressive (biting or locking mandibles). An additional behaviour classified as peaceful was 'mounting', where the smaller *Crematogaster *(body length approx. 2–3 mm) climbed onto the *Camponotus *body (body length 5–13 mm). Continued interactions were recorded again after 10 s (the same behavioural classification as used in [2]).

The aggressiveness of two other *Cr. modiglianii *colonies was estimated in arena confrontations. The workers had been collected in the forest one day prior to the tests and were kept in a plastic box among nestmates (but separate from the partner species) over night. Five *Cr. modiglianii *individuals were placed into a fluon-covered plastic cylinder (Ø 7.5 cm, height 5 cm) on top of a paper sheet floor. After 1 min to calm down, a dead *Camponotus *specimen was introduced. For the following 100 s we recorded the behaviour of the ants as above. Each living or dead ant was used for one assay only. In all of the above assays, we performed ten replicates per treatment.

From each replicate we calculated the proportions of all aggressive versus all non-aggressive interactions. Both strong and total (including weak) aggression were analyzed using generalized linear models (GLM) with quasibinomial error distribution and logit link function. In order to determine whether confrontations within and across chemical varieties differ, we used the according explanatory variable 'within/across variety' with two factor levels (which collapsed to '*Camponotus *variety' in the first dataset). The variable 'variety combination' (with the factor levels 'black→black', 'black→red', 'red→red', and 'red→black') was nested in the former one. Further explanatory variables were 'colony combination' (nested in 'variety combination'), which collapsed to '*Camponotus *colony' in the first dataset, and 'intra-/allocolonial'. Due to their nested structure, no interactions between the variables were possible. The impact of each variable was determined by likelihood ratio tests (F tests). We also re-analyzed data from [2] in a similar way, where we included the number of workers present in the experimental arena as explanatory variables (see [2] for details on the experimental setup). Since the statistical results for total aggression and for strong aggression only were similar, only the former will be reported in the results section. All computations were performed in R Version 2.5.1 [55].

## Competing interests

The authors declare that they have no competing interests.

## Authors' contributions

NB conceived the study and designed the experimental setup. FM collected the samples, performed the quantitative analysis of the cuticular substances, the behavioural experiments and the statistical analyses and wrote the manuscript. TS contributed significantly to the concept and the design of the study and identified the cuticular substances. Both NB and TS contributed to the preparation of the manuscript. All authors read and approved the final manuscript.
